# The impact of nurse-led care in inflammatory bowel disease management: a systematic review

**DOI:** 10.1186/s12912-026-04678-w

**Published:** 2026-04-27

**Authors:** Xiaoran Wang, Nan Zhang, Xiaotong LI, Qiaomei Zhang, Yandi Liu, Hongwen Ma

**Affiliations:** 1 Department of Gastroenterology, Tianjin Union Medical Center, The First Affiliated Hospital of Nankai University, No.190 Jieyuan Road, Hongqiao District, Tianjin, 300121 P. R. China; 2 Department of Nursing, Tianjin Union Medical Center, The First Affiliated Hospital of Nankai University, No.190 Jieyuan Road, Hongqiao District, Tianjin, 300121 P. R. China

**Keywords:** Chronic disease management, Inflammatory bowel disease, Nurse-led care, Quality of life, Self-efficacy, Systematic review

## Abstract

**Background:**

Inflammatory Bowel Disease (IBD), which includes Crohn’s disease and ulcerative colitis, is a chronic disease that requires long-term, coordinated management. Although nurse-led care is a pivotal element of effective IBD management through specific interventions, a comprehensive synthesis of its overall impact is lacking in the literature.

**Aim:**

To review systematically and synthesize evidence on the effectiveness of nurse-led care compared to usual care for individuals with IBD.

**Methods:**

A systematic search of PubMed, Web of Science, Scopus, Cochrane Library, and Embase was conducted for Randomized controlled trials (RCTs) and observational studies (cohort or case-control studies) published from database inception to April 30, 2025. The methodological quality of included studies was assessed using the critical appraisal tools from the Joanna Briggs Institute (JBI), and this review adhered to the Preferred Reporting Items for Systematic Reviews and Meta-Analyses (PRISMA) guidelines.

**Results:**

Sixteen studies with a total of 2,468 participants were included. Nurse-led care significantly improved QoL (9 of 16 studies), self-management (5 of 6 studies), and reduced anxiety/depression/disease uncertainty (8 of 10 studies). It also improved sleep quality (4 of 4 studies), and patient satisfaction (2 of 2 studies). However, none of the 8 studies reported significant differences in disease activity between the groups, and no significant differences were found for smoking cessation motivation, coping capacity, medication adherence, or patient concerns. Structured, high-intensity interventions demonstrate benefits, whereas brief approaches show limited effectiveness. Outcome variability suggests clinical and methodological heterogeneity, underscoring the need for more rigorously designed studies.

**Conclusions:**

Nurse-led care has the potential to improve psychosocial well-being, QoL, and self-management capabilities in patients with IBD, though the realization of these benefits requires healthcare systems to implement structured, high-intensity programs that move beyond simple advisory roles. Future research should therefore prioritize investigating the long-term sustainability of benefits, cost-effectiveness, and hybrid care models that integrate nursing support with medical management to determine their potential impact on disease activity.

**Clinical trial number:**

Not applicable.

**Supplementary Information:**

The online version contains supplementary material available at 10.1186/s12912-026-04678-w.

## Introduction

Inflammatory bowel disease (IBD), which includes Crohn’s disease and ulcerative colitis, is a chronic and relapsing disorder characterized by intestinal inflammation. It affects over 6.8 million people globally [1], yet etiology is not fully elucidated [2]. IBD is associated with severe complications that impose a significant physical, functional, and psychological burden on patients [3]. This profoundly impairs their quality of life (QoL) and also creates great burdens for healthcare systems and socioeconomic productivity [4, 5].

Rapid economic development and lifestyle changes have driven a global increase in the prevalence of IBD, which is no longer confined to Western nations but has also begun to rise in newly industrialized countries [6, 7]. Forecasts indicate that IBD prevalence will reach 1.1% in Canada by 2035 [8], and 1.02% in Lothian, Scotland by 2028, for example [9]. Many newly industrialized regions have undergone an “acceleration in incidence,” characterized by a swift growth in both IBD incidence and prevalence [4, 10]. This trend, combined with higher care costs, has placed unsustainable strain on healthcare systems [11] and underscores a pressing need for integrated and holistic management strategies [12]. Consequently, nurse-led care has become a crucial component of IBD management by providing essential support through patient education, symptom monitoring, and psychosocial care.

A web-based survey of 4,670 patients from 25 European countries revealed that 48% reported insufficient access to care [13]. Despite expanding care options and substantial resources allocated to IBD management, a number of patients still failed to achieve their therapeutic goals [14]. These practice variations reflect persistent gaps in healthcare delivery and can lead to adverse outcomes, such as increased reliance on acute care, higher surgical rates, reduced work prolificacy, and diminished QoL [15].

The complexity of IBD management necessitates a multidisciplinary approach. Indeed, such an approach has been found to enhance clinical outcomes, optimize resource utilization, reduce hospital admissions [16, 17], and improve mental health and patient satisfaction [18]. Within their care teams, nurses play an indispensable role by contributing to both the clinical and managerial dimensions of care [19]. Consequently, there is growing consensus to expand the role of specialist IBD nurses within modern multidisciplinary services, alongside calls for increased funding and staffing [17]. As frontline coordinators, they facilitate patient self-management by providing evidence-based education and sustained support [20]. IBD nurses, who work in accordance with clinical guidelines, possess in-depth knowledge of disease pathophysiology and demonstrate advanced competencies in patient education, care coordination, and research [21].

Despite the fact that the role of nurses in IBD management is widely believed to be beneficial, the role lacks a standardized definition, and empirical evidence supporting nurses’ efficacy remains fragmented. Although several systematic reviews have explored the role of nurses in IBD management [22, 23], this growing body of work often overlooks the full spectrum of relevant outcomes, including objective clinical metrics and patient-reported experiences. Moreover, factors that influence intervention success have not been adequately analyzed, such as intensity, delivery mode, and theoretical foundation. Therefore, further study is needful to clarify the impact of nurse-led care and to develop more robust, normative, and systematic care models.

This study and others like it are urgently needed because without a clear understanding of these active components, healthcare systems struggle to implement, standardize, and scale nurse-led care models effectively. Although the efficacy of nurse-led care has been established in other chronic diseases, the evidence specific to IBD is fragmented. Therefore, a comprehensive and updated synthesis is urgently required to clarify the overall impact of nurse-led care and to guide its effective implementation.

This systematic review was thus undertaken in order to synthesize the current literature on the effectiveness of nurse-led care versus usual care for adults with IBD. Specifically, we evaluate its impact on a broad range of health outcomes, including QoL, self-management, psychological health, disease activity, and other health outcomes. The review also examines key interventions, such as typology, duration, and measurement tools, and discusses implications for evidence-based nursing practice. Furthermore, it seeks to identify the core components of successful interventions, evaluate the methodological quality of existing studies, and highlight priorities for future research.

## Methods

This study was designed as a systematic review and carried out in accordance with the Preferred Reporting Items for Systematic Reviews and Meta - Analyses (PRISMA) 2020 statement [24].The purpose of this design was to evaluate and synthesize evidence regarding the specific effectiveness of nurse-led care on the health outcomes of adults with IBD as rigorously as possible. Several stages of the study were executed, including the search and screening, data extraction, quality assessment, and result synthesis.

### Search strategy

An overall evidence search was performed on May 30, 2025 of the following five databases: PubMed, Web of Science, Scopus, Cochrane Library, and Embase. These databases were selected to ensure a broad coverage of biomedical, nursing, and interdisciplinary literature, thereby minimizing the risk of publication bias and ensuring the capture of both clinical trials and observational studies. The search strategy combined Medical Subject Headings (MeSH) and free-text terms related to the populations studied (e.g. “Inflammatory Bowel Diseases”, “Crohn Disease”, “Colitis, Ulcerative”) as well as and the interventions they received (e.g. “Practice Patterns, Nurses”, “Nurse-Led”, “Nursing Care”). Boolean operators (AND, OR) were used to refine the search. Specific search strings are detailed in the supplementary material.

### Protocol registration

The review protocol was registered a priori in the PROSPERO database (Registration number: CRD420251002506).

### Inclusion and exclusion criteria

Nurse-led care was operationally defined as holistic interventions in which nurses exercised professional autonomy in patient management, and it ranged from structured education to independent nonpharmacological symptom management. The inclusion criteria were determined according to the PICOS principle, as shown below: (a) population: adults (≥ 18 years) diagnosed with IBD (Crohn’s disease or ulcerative colitis), (b) intervention: any nurse-led practice pattern or clinic for IBD management, in any setting or country, (c) comparison: usual care, routine care, or no intervention, (d) outcome: QoL and other health outcomes. (e) study design: randomized controlled trials (RCTs) and observational studies (cohort or case-control designs). The following exclusion criteria were applied: (a) non-English publications, (b) Studies focusing on pediatric populations, (c) reviews, conference abstracts, letters, or guidelines, (d) and studies without accessible full text.

Nurse-led care was operationally defined as holistic interventions where nurses exercised professional autonomy in patient management, ranging from structured education to independent non-pharmacological symptom management.

### Selection process and data extraction

After removing duplicates using EndNote software, two reviewers (XRW and QMZ) independently screened titles, abstracts, and full texts of studies according to eligibility criteria. References of included articles were also hand-searched for additional relevant studies. Any discrepancies were discussed with a third reviewer (HWM). The reference lists of all included articles were also hand-searched to identify additional relevant studies. Data were extracted using a standardized form in Microsoft Excel (Microsoft Corp, USA), Two authors (XTL and NZ) independently extracted the following data items: (1) study characteristics (author, year, country, design); (2) participant details (sample size, mean age, disease type); (3) intervention specifics (type, duration, frequency, delivery method); and (4) outcomes measured and key findings. All inconsistencies between these two were resolved by a third reviewer (YDL).

### Quality assessment

Because a meta-analysis could not be performed due to high heterogeneity, methodological quality of included studies was evaluated using the Joanna Briggs Institute (JBI) Critical Appraisal Checklists [25]. Two reviewers independently assessed each study. RCTs were evaluated using a 13-item checklist, and cohort studies with an 11-item checklist. Items were scored as “yes” (1 point), “no” (0 points), “unclear”, or “not applicable”. Based on the percentage of “yes” scores, studies were classified as high quality (≥ 67%), medium quality (34–66%), or low quality (≤ 33%).

### Data synthesis

Due to heterogeneity in intervention designs (ranging from RCTs to observational studies), follow-up duration (ranging from 4 weeks to 12 months, resulting in insufficient studies at any single common timepoint to allow for meaningful pooling), and outcome measures (e.g. use of different QoL scales like IBDQ vs. SF-36), a narrative synthesis was performed. To minimize repetitive reporting caused by the diverse nature of the interventions, findings were organized by outcome domain and summarized descriptively. This process involved three steps: (1) extracting and tabulating key descriptive data to identify similarities and differences across studies; (2) grouping the findings by primary outcome domains (e.g. QoL, disease activity, self-management); and (3) analyzing how intervention characteristics may explain variations in the observed outcomes.

## Results

### Study selection

**Fig. 1:**
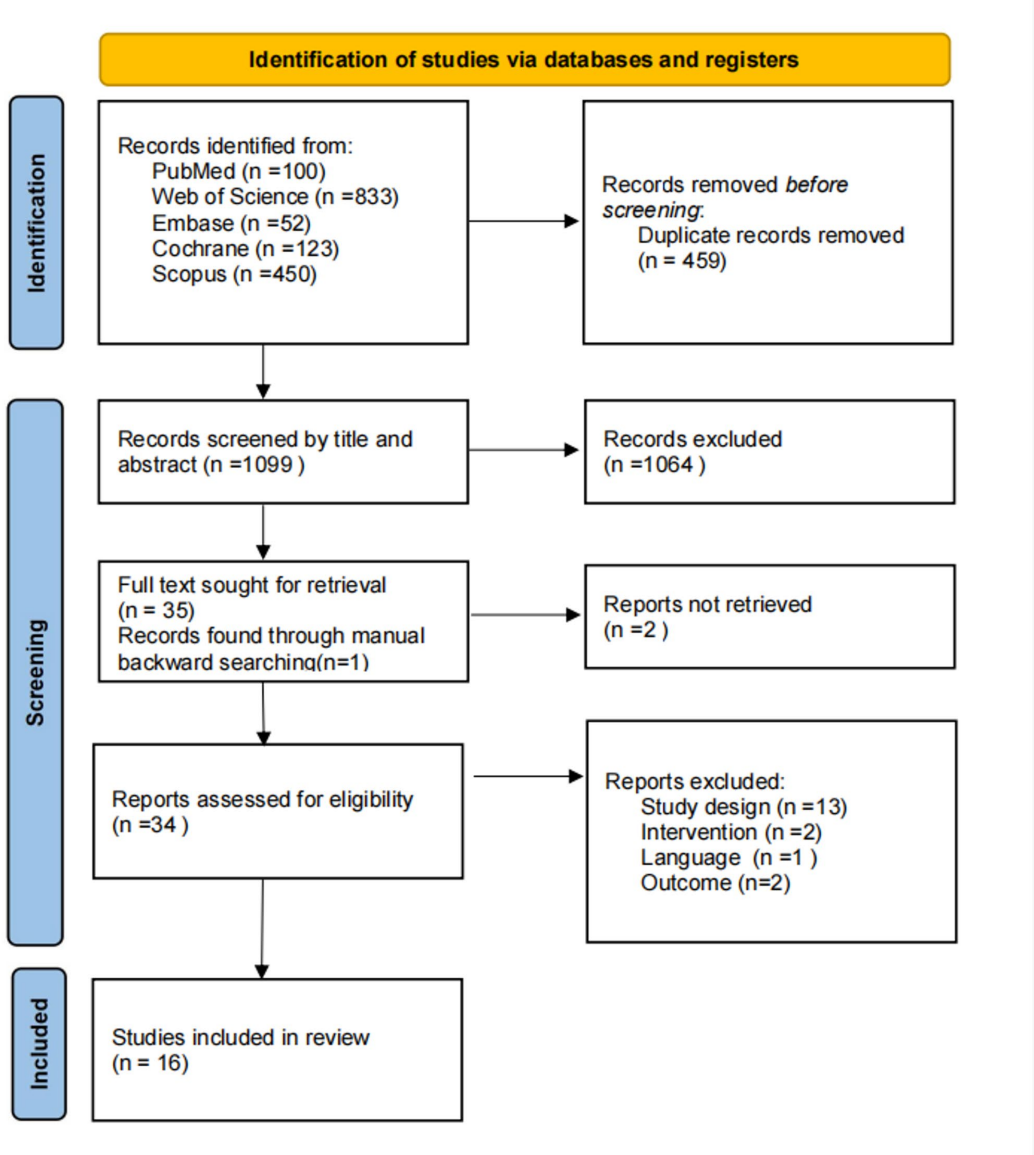
PRISMA flowchart for study screening

One thousand five hundred eighty-eight articles were initially identified from the five databases. The detailed study screening and selection process is illustrated in the PRISMA flowchart (Fig. 1). After the elimination of 459 duplicate records, a total of 1,099 studies were subjected to title and abstract screening based on the inclusion and exclusion criteria. This led to the exclusion of 1,064 articles. Subsequent assessment of the full text was performed for the remaining 33 articles, as two reports could not be retrieved. The backward snowballing technique, which involved manually searching reference lists, identified one additional relevant study. A total of 34 studies were thus assessed for quality, of which 18 were excluded. Ultimately, this systematic review covered 16 articles.

### Quality of included studies

The methodological quality and specific risk of bias for each included study are detailed in Table 1. Based on the proportion of the total possible score achieved, study quality was categorized into three levels: high quality (≥ 2/3 of total score), medium quality (> 1/3 but < 2/3), or low quality (≤ 1/3). Among the 16 studies, 10 were rated as high quality and 6 as medium quality.

**Table 1:** Critical appraisal

Study	Study design	Checklist tool	JBI critical appraisal
Zhou, 2024	RCTs	Checklist for RCTs	8/13(61.54%)
Bokemeyer, 2024	RCTs	Checklist for RCTs	8/13(61.54%)
You, 2022	RCTs	Checklist for RCTs	9/13(69.23%)
Xi, 2022	RCTs	Checklist for RCTs	7/13(53.85%)
Navarro Correal, 2021	RCTs	Checklist for RCTs	9/13(69.23%)
Magharei, 2019	RCTs	Checklist for RCTs	9/13(69.23%)
Del Hoyo, 2018	RCTs	Checklist for RCTs	10/13(76.92%)
Jaghult, 2007	RCTs	Checklist for RCTs	9/13(69.23%)
Oxelmark, 2007	RCTs	Checklist for RCTs	7/13(53.85%)
Smith, 2002	RCTs	Checklist for RCTs	9/13(69.23%)
Shi, 2024	Cohort	Checklist for Cohort Studies	7/11(63.64%)
Mercuri, 2024	Cohort	Checklist for Cohort Studies	10/11(90.91%)
Huang, 2024	Cohort	Checklist for Cohort Studies	9/11(81.82%)
Yu, 2023	Cohort	Checklist for Cohort Studies	6/11(54.55%)
Xu, 2023	Cohort	Checklist for Cohort Studies	9/11(81.82%)
Jelsness-Jorgensen, 2012	Cohort	Checklist for Cohort Studies	10/11(90.91%)

### Characteristics of included studies

The 16 included studies (10 RCTs and 6 Cohort Studies), published between 2002 and 2024, involved a total of 2,468 participants from eight countries. Among the included studies, one was from Germany [26], two were from Spain [27, 28], two were from Iran [29], two were from Sweden [30, 31], one was from the United Kingdom [32], one was from Italy [33], one was from Norway [34], and the rest were from China. The sample sizes ranged from 40 to 1,066. Furthermore, nurse-led interventions varied widely in design, duration, and delivery mode. The most frequently assessed outcome was QoL, primarily measured using the Inflammatory Bowel Disease Questionnaire (IBDQ) or its short forms (sIBDQ or IBDQ-9), and intervention durations ranged from 7 days to 18 months. The key characteristics, intervention components, and clinical outcomes of the 16 included studies are summarized in Table 2.

**Table 2:** Characteristics of Included Studies

Study ID (Author, Year)	Country	Sample Size (I/C)	Intervention Description	Duration	Primary Outcomes
Bokemeyer, 2024	Germany	1066 (540/526)	I: Quarterly consultations with trained IBD nurse. C: Usual care.	18 months	Disease-specific QoL (-) Generic QoL (-) Disease activity (-)
Del Hoyo, 2018	Spain	42 (21/21)	I: Nurse-assisted telephone care. C: Standard in-person visits.	24 weeks	Disease Activity ↓ QoL (-) Medication Adherence (-) Satisfaction (-)
Huang, 2024	China	275 (151/124)	I: Ward noise-reduction management. C: Routine care.	7 days	Anxiety ↓ Insomnia ↓ QoL ↑
Jaghult, 2007	Sweden	84 (52/32)	I: Multiprofessional education program. C: Regular information.	6 months	General health (-) Coping capacity (-) QoL (-) Worries (-)
Jelsness-Jorgensen, 2012	Norway	140 (71/69)	I: Specialist IBD nurse clinic C: Conventional follow-up.	1 year	Worries (-) QoL (-)
Magharei, 2019	Iran	64 (32/32)	I: 6-session education program & 1-month phone follow-up. C: Routine care.	3 weeks	Self-efficacy ↑ QoL ↑
Mercuri, 2024	Italy	50 (26/24)	I: Regular nurse telephone/email support. C: Standard care.	6 months	Anxiety ↓ Depression ↓ Fatigue ↓ Social satisfaction ↑ Sleep quality ↑ QoL ↑
Navarro Correal,2021	Spain	144(72/72)	I: 5 R’s model motivational intervention by IBD nurses. C: Regular care	1 year	Motivation to quit (-) Stage of change (-) Smoking status (-) Nicotine dependence (-)
Oxelmark,2007	Sweden	44 (24/20)	I: 9-week didactic and interactive group therapy. C: Regular information	12 months	QoL (-) Coping capacity (-) Overall health ↑
Shi, 2024	China	112 (50/62)	I: CITM-based nursing. C: Routine care.	3 months	Symptom scores ↓ Anxiety ↓ Depression ↓ Self-care ability ↑ QoL ↑
Smith, 2002	UK	100 (50/50)	I: Nurse-led counselling package. C: Routine clinical follow-up.	12 months	Disease Activity (-) Mean Mental Health ↑ Anxiety (-) Depression (-)
Xi, 2022	China	40 (20/20)	I: Empowerment education and mindfulness meditation. C: Conventional nursing.	3 months	Anxiety ↓ Medication compliance ↑ Life compliance ↑ QoL ↑
Xu, 2023	China	97 (49/48)	I: Continuous nursing model. C: Routine nursing.	6 months	Disease uncertainty ↓ Medical coping style ↑ Sleep quality ↑ QoL ↑
You, 2022	China	70 (35/35)	I: Aromatherapy (skin/inhalation). C: Routine nursing.	8 weeks	Fatigue ↓ Sleep quality ↑ QoL ↑
Yu, 2023	China	80 (43/37)	I: Hospital-family holistic continuing care. C: Routine care.	6 months	Anxiety ↓ Depression ↓ Nutrition status ↑ Knowledge ↑ Self-efficacy ↑ Satisfaction ↑ QoL ↑
Zhou, 2024	China	60 (30/30)	I: WeChat-based continuous care and empowerment education. C: Routine care.	3 months	Disease uncertainty ↓ Hope level ↑ Self-care Ability ↑ Patient satisfaction ↑ QoL ↑

Note: I = Intervention; C = Control; IBD = Inflammatory Bowel Disease; QoL = Quality of Life; CITM = Chronic Illness Trajectory Model. (↑) = Significant Improvement; (↓) = Significant Reduction; (-) = No Significant Difference

### Effectiveness of nurse-led interventions

The synthesis of our findings is presented by key outcome domains below. Based on the detailed data extracted from the selected studies, a comprehensive understanding of the impact of nurse-led care in IBD management emerged across several of them.

### QoL

Nine of the sixteen studies (56.3%) reported statistically significant improvements in disease-specific QoL following nurse-led interventions. Effective interventions were typically high in intensity and structure, such as continuous WeChat-supported multidisciplinary care [35], multi-session empowerment education [29], and integrated hospital-family holistic models [36]. For instance, Shi and Geng (2024) reported significantly superior IBDQ scores in the nurse-led cohort at 12 weeks compared to the control group (178.03 ± 4.64 vs. 163.41 ± 4.46, *P* < 0.05). Significant QoL gains were also documented by You et al. (2022) at 12 weeks (164.60 ± 17.00 vs. 145.71 ± 18.30, *P* < 0.05) and Magharei et al. (2019) at 4 weeks (44.69 ± 5.04 vs. 38.56 ± 6.27, *P* < 0.05). Furthermore, Yu and Guo (2023) [36] observed a significant increase in IBDQ scores after 6 months of continuous care (*P* < 0.001). In contrast, seven studies (43.8%) found no significant intergroup differences in QoL, which were generally associated with lower-intensity interventions, such as quarterly consultations [26] or short-term group therapy [30, 31].

### Self-efficacy and self-care ability

Five out of six studies (83.3%) reported significant improvements in self-efficacy and self-care ability. Structured educational programs, such as the six-session intervention by Magharei et al. [29], led to notable increases in self-efficacy scores (*P* < 0.001), which were positively correlated with improved QoL (*r* = 0.32, *P =* 0.01). Empowerment-based approaches [35] and chronic illness trajectory models [37] also showed marked benefits in self-care ability (*P <* 0.05).

### Psychosocial outcomes

Significant reductions in anxiety and depression were observed in 8 out of 10 studies (80%). Effective strategies included continuous nursing models [38], mindfulness meditation [39], and remote telephone support [29]. For example, Mercuri et al. [33] reported substantial reductions in both anxiety (*P <* 0.001) and depression (*P <* 0.0001) after six months of telecare. Illness-related uncertainty was also significantly reduced in interventions such as the WeChat-based support program by Zhou et al. [35] (*P <* 0.05). An exception was a smoking cessation intervention using motivational interviewing, which showed no significant effect on motivation or behavioral change [27].

### Sleep quality

All four studies (100%) that assessed sleep quality reported significant improvements. Interventions such as aromatherapy [40] and noise-reduction [41] management in wards were particularly effective. For instance, Huang et al. [36] noted significantly lower insomnia scores in the intervention group (*P <* 0.05), and Mercuri et al. [33] also observed improved sleep quality (*P =* 0.0013) following remote support.

### Disease activity

None of the eight studies that evaluated disease activity using standardized indices found significant differences between nurse-led interventions and usual care. Large-scale trials, such as that by Bokemeyer et al. [26] (*n* = 1,066), also found no intergroup differences at any assessment point. Although one study reported a trend toward improvement (*P =* 0.10) [28], the collective evidence indicates that current nurse-led models do not significantly alter objective disease activity.

### Other outcomes

Notable improvements were observed in several secondary outcomes. Fatigue was significantly reduced through aromatherapy [40] and structured remote support [33] (*P* < 0.05). Educational interventions also promoted better health behaviors. For example, empowerment education combined with mindfulness training resulted in higher medication and lifestyle compliance (*P* < 0.05) [39]. A hospital-family integrated care model improved nutritional status, reflected in improved MNA and SGA scores, and increased prealbumin and albumin levels (*P* < 0.001) [36]. However, a smoking cessation program based on the 5 R’s model showed no significant differences in quit rates or nicotine dependence despite a higher proportion of quitters in the intervention group (20.9% vs. 13.2%) [27].

### Intervention intensity and duration

Interventions characterized by higher intensity, longer duration (≥ 3 months), and structured multi-session delivery generally appeared to be associated with more favorable outcomes across psychosocial and self-management domains. In contrast, brief, low-intensity consultations more frequently reported limited effectiveness.

## Discussion

This study synthesized evidence from 16 studies to evaluate the impact of nurse-led care on QoL and other health outcomes in patients with IBD. The included interventions were diverse, with many incorporating non-pharmacological strategies such as aromatherapy and noise reduction. The findings indicate that nurse-led interventions provide significant and clinically meaningful benefits, particularly in psychosocial and QoL domains. However, these results must be interpreted with caution due to the observed heterogeneity, only a slight majority of studies (9 out of 16) reported statistically significant improvements in QoL. The heterogeneity in outcomes found in our analysis, particularly for QoL, can be partly explained by differences in intervention intensity and core components. Moreover, the neutral findings from several European RCTs [26, 30] may be attributable to their reliance of low-intensity interventions, such as quarterly consultations. These low-intensity approaches contrast sharply with the high-intensity, multi-session educational programs [29] and continuous remote monitoring strategies [33] that showed positive results. Interventions focused solely on providing information were less effective than those that incorporated skill-building, behavioral strategies [39], or ongoing motivational support. Furthermore, Multiple factors modulate the QoL of patients with IBD. Adverse psychological states [42, 43], smoking, hospitalization, corticosteroid use [44], and disruptions to daily activities and social interactions [45] are all negatively associated with QoL and reduced life satisfaction [46].

This variability indicates that the mere presence of a nurse is insufficient to guarantee improved outcomes; rather, the success of an intervention appears to depend heavily on its structure, content, and intensity. Interventions that were structured, high-intensity, longer duration (≥ 3 months), and higher frequency (≥ 4 sessions) significantly more effective. These findings are consistent with and extend the existing evidence base for chronic disease management. Successful interventions often incorporated high-frequency interactions and were grounded in empowerment theory [29, 35, 39]. The significant positive impact on psychosocial outcomes in particular underscores a distinct advantage of nursing interventions: their capacity to address the critical biopsychosocial aspects of IBD care [47]. This indicates that enhancing patients’ knowledge, skills, and confidence is a key mechanism by which nursing care improves health outcomes [48]. The success of technology-enabled platforms underscores the growing importance of digital health. It demonstrates how nurses can leverage technology to overcome geographic and temporal barriers, thereby providing sustained support, a finding consistent with earlier reviews of digital health in IBD [49].

The lack of significant improvement in disease activity indices is an important finding and suggests that although nurse-led care excels in modifying the behavioral and biopsychosocial dimensions of the illness, its direct effect on the underlying inflammatory process may be limited. Nurses are uniquely positioned to enhance medication adherence, facilitate lifestyle modifications, improve patients’ coping strategies for symptom burden, and provide early triage during potential flares. This distinction indicates that nurse-led interventions should serve as a complement to, not a replacement for, pharmacologic and medical management within an integrated, multidisciplinary care model [50, 51]. Further research is therefore needed to clarify nurse role in both disease activity and progression.

The specific role of nurses in IBD management is continually evolving. IBD nurse specialists are increasingly recognized valuable and cost-effective [52], and they provide accessible advice, facilitate outpatient management, and reduce hospital admissions [53, 54]. In one survey, over 87% of respondents believed that nurses should identify the need for referrals to other professionals to optimize patient well-being [3]. Indeed, large-scale quality improvement initiatives have successfully implemented nurse-led telephone clinics as the core of stratified IBD management systems. These clinics were able to achieve noninferior patient satisfaction levels compared to standard care while also enhancing care responsiveness and sustainability [55]. Telephone-based nursing services are particularly impactful. For instance, nurse-managed advice lines proactively address most disease-related concerns [56] and have proven to be both feasible and well-accepted among patients with stable IBD [57]. One study demonstrated that telephone care is more cost-effective than standard follow-up [58]. More broadly, telemedicine promotes self-management and treatment optimization through remote monitoring and education [59, 60]. However, the unique contribution of nurses within these telemedicine frameworks remains poorly defined. This gap highlights a critical need for future research to define precisely and optimize their role within digital health models.

### Implications of the study

This review highlights the potential value of nurse-led care in managing IBD. It confirms the essential role of nurses in delivering high-value interventions, including continuous remote support, empowerment-based education, and integrated care models. The integration of structured, nurse-led initiatives into standard multidisciplinary IBD care should be considered by healthcare systems, especially in outpatient and community settings. Future research must aim to identify the core active components of effective interventions and address the current limitations in evidence quality. This requires the use of standardized, high-fidelity protocols and comprehensive reporting to facilitate replication and allow for meaningful cross-study comparisons. This requires the use of standardized, high-fidelity protocols and comprehensive reporting to facilitate replication and allow for meaningful cross-study comparisons. Furthermore, future investigations should focus on long-term outcomes, cost-effectiveness, and blended care models that seamlessly integrate nursing support with medical management.

### Study limitations

Several limitations of this review should be acknowledged. First, considerable clinical and methodological heterogeneity in intervention designs, outcome measures, and follow-up durations precluded a meta-analysis and necessitated a narrative synthesis. Second, despite employing a comprehensive search strategy, the limit to English-language publications may have introduced language bias by omitting relevant studies published in other languages. Third, the substantial variability in intervention intensity, duration, and the degree of nurse involvement across the included studies limits the comparability of their outcomes. Furthermore, while the overall study quality is moderate to high, cautious interpretation is necessary due to potential bias arising from inadequate descriptions of allocation concealment, incomplete reporting of randomization methods, and a lack of detail regarding participant dropouts. Finally, the long-term sustainability of the observed benefits remains unclear, as the majority of studies had follow-up periods of six months or less.

## Conclusion

This systematic review provides robust evidence that nurse-led care can be beneficial for patients with IBD, particularly psychosocial well-being and QoL, in individuals with IBD. Nurses play an indispensable role in chronic disease management by providing continuous, empathetic, and personalized care through various channels, including in-person consultations, remote support, and structured education. To optimize health outcomes and ensure the equitable utilization of this beneficial model, future initiatives must pay close attention to three priorities: standardizing the core components of nurse-led IBD care, tailoring interventions to individual patient needs, and expanding self-management education programs. As healthcare systems increasingly prioritize value-based and integrated care, nurse-led models are poised to become essential for delivering comprehensive, high-quality, and scalable IBD management across diverse settings.

## Supplementary Information

Below is the link to the electronic supplementary material.

Supplementary Material 1

## Data Availability

No datasets were generated or analysed during the current study.
