# Supplementary material for: The impact of nurse-led care in inflammatory bowel disease management: a systematic review
Source: BMC Nurs. 2026 Apr 27;25:522. doi: 10.1186/s12912-026-04678-w (PMC13251159; doi:10.1186/s12912-026-04678-w)
Supplement: Supplementary file 1 — Supplementary Material 1 [file 12912_2026_4678_MOESM1_ESM.docx]

SEARCH STRINGS

## PUBMED: 100 articles

**Search Performed on:** May 30, 2025

((((((((((((((((((((((((Inflammatory Bowel Diseases[MeSH Terms]) OR (Inflammatory Bowel Disease[Title/Abstract])) OR (Bowel Diseases, Inflammatory[Title/Abstract])) OR (Colitis, Ulcerative[MeSH Terms])) OR (Colitis Gravis[Title/Abstract])) OR (Idiopathic Proctocolitis[Title/Abstract])) OR (Inflammatory Bowel Disease, Ulcerative Colitis Type[Title/Abstract])) OR (Ulcerative Colitis[Title/Abstract])) OR (Crohn Disease[MeSH Terms])) OR (Crohn's Disease[Title/Abstract])) OR (Crohns Disease[Title/Abstract])) OR (Crohn's Enteritis[Title/Abstract])) OR (Inflammatory Bowel Disease 1[Title/Abstract])) OR (Regional Enteritis[Title/Abstract])) OR (Ileocolitis[Title/Abstract])) OR (Ileitis, Terminal[Title/Abstract])) OR (Terminal Ileitis[Title/Abstract])) OR (Ileitis, Regional[Title/Abstract])) OR (Regional Ileitis[Title/Abstract])) OR (Enteritis, Granulomatous[Title/Abstract])) OR (Granulomatous Enteritis[Title/Abstract])) OR (Enteritis, Regional[Title/Abstract])) OR (Colitis, Granulomatous[Title/Abstract])) OR (Granulomatous Colitis[Title/Abstract])) AND (((((Nursing Care[MeSH Terms]) OR (Care, Nursing[Title/Abstract])) OR (Management, Nursing Care[Title/Abstract])) OR (Nursing Care Management[Title/Abstract])) OR (((((((((((((Practice Patterns, Nurses'[MeSH Terms]) OR (Nurses' Practice Patterns[Title/Abstract])) OR (Nurse's Practice Patterns[Title/Abstract])) OR (Nurse Practice Patterns[Title/Abstract])) OR (Nurse's Practice Pattern[Title/Abstract])) OR (Practice Pattern, Nurse's[Title/Abstract])) OR (Practice Patterns, Nurse's[Title/Abstract])) OR (Nurse-Led Clinics[Title/Abstract])) OR (Clinic, Nurse-Led[Title/Abstract])) OR (Clinics, Nurse-Led[Title/Abstract])) OR (Nurse-Led Clinic[Title/Abstract])) OR (Nurse Led Clinics[Title/Abstract])) OR (Nurse Intervention[Title/Abstract])))

## Web of Science: 833 articles

**Search Performed on:** May 30, 2025

**#1** (((((((((((((((TS=(Inflammatory Bowel Diseases)) OR TS=(Colitis, Ulcerative)) OR TS=( Colitis Gravis)) OR TS=(Idiopathic Proctocolitis)) OR TS=(Inflammatory Bowel Disease, Ulcerative Colitis Type)) OR TS=(Crohn Disease)) OR TS=(Crohn's Disease)) OR TS=(Crohns Disease)) OR TS=(Crohn's Enteritis)) OR TS=(Inflammatory Bowel Disease 1)) OR TS=(Regional Enteritis)) OR TS=(Ileocolitis)) OR TS=(Terminal Ileitis)) OR TS=(Regional Ileitis)) OR TS=(Granulomatous Enteritis)) OR TS=(Granulomatous Colitis)

**#2** (((((((((TS=(Nurses' Practice Patterns)) OR TS=(Nurse's Practice Patterns)) OR TS=(Nurse Practice Patterns)) OR TS=(Nurse's Practice Pattern)) OR TS=(Nurse-Led Clinics)) OR TS=(Nurse-Led Clinic)) OR TS=(Nurse Led Clinics)) OR TS=(Nursing Care)) OR TS=(Nursing Care Management)) OR TS=(Nurse Intervention)

**#3** #2 AND #1 and Preprint Citation Index (Exclude – Database) and Article (Document Types)

## Embase: 52 articles

Search Performed on: May 30, 2025

**#1** 'inflammatory bowel diseases'/exp OR 'inflammatory bowel disease'/exp OR 'chronic ulcerative colitis'/exp OR 'colitis ulcerativa'/exp OR 'colitis ulcerosa'/exp OR 'colitis ulcerosa chronica'/exp OR 'colitis, mucosal'/exp OR 'colitis, ulcerative'/exp OR 'colitis, ulcerous'/exp OR 'colon, chronic ulceration'/exp OR 'histiocytic ulcerative colitis'/exp OR 'mucosal colitis'/exp OR 'ulcerative colorectitis'/exp OR 'ulcerative procto colitis'/exp OR 'ulcerative proctocolitis'/exp OR 'ulcerous colitis'/exp OR 'ulcerative colitis'/exp OR 'cleron disease'/exp OR 'Crohn`s disease'/exp OR 'Crohns disease'/exp OR 'enteritis regionalis'/exp OR 'intestinal tract, regional enteritis'/exp OR 'morbus crohn'/exp OR 'regional enteritis'/exp OR 'regional enterocolitis'/exp OR 'Crohn disease'/exp

**#2** 'nursing care'/exp OR 'NP practice patterns'/exp OR 'nurse practice patterns'/exp OR 'nurse`s practice patterns'/exp OR 'nurses practice patterns'/exp OR 'practice patterns, nurses`'/exp OR 'nursing practice'/exp

**#3= #1 AND #2**

## Cochrane: 123 articles

**Search Performed on:** May 30, 2025

Search Name:

Date Run: 30/05/2025 01:54:37

Comment:

ID Search Hits

#1 MeSH descriptor: [Inflammatory Bowel Diseases] explode all trees 5035

#2 (Bowel Diseases, Inflammatory OR Inflammatory Bowel Disease):ti,ab,kw 5392

#3 MeSH descriptor: [Colitis, Ulcerative] explode all trees 2324

#4 (Idiopathic Proctocolitis OR Inflammatory Bowel Disease, Ulcerative Colitis Type OR Ulcerative Colitis OR Colitis Gravis):ti,ab,kw 7150

#5 MeSH descriptor: [Crohn Disease] explode all trees 2340

#6 (Ileocolitis OR Regional Ileitides OR Ileitis, Regional OR Terminal Ileitis OR Regional Ileitis OR Ileitis, Terminal OR Enteritis, Granulomatous OR Enteritis, Regional OR Granulomatous Enteritis OR Crohn's Disease OR Crohn's Enteritis OR Inflammatory Bowel Disease 1 OR Regional Enteritis OR Crohns Disease OR Granulomatous Colitis OR Colitis, Granulomatous):ti,ab,kw 8629

#7 #1 OR #2 OR #3 OR #4 OR #5 OR #6 14317

#8 MeSH descriptor: [Practice Patterns, Nurses'] explode all trees 202

#9 (Nurse-Led Clinic OR Nurse Led Clinics OR Nurse-Led Clinics OR Clinics, Nurse-Led OR Clinic, Nurse-Led OR Nurses' Practice Patterns OR Nurse's Practice Pattern OR Nurse Practice Patterns OR Practice Pattern, Nurse's OR Nurse's Practice Patterns OR Practice Patterns, Nurse's):ti,ab,kw 1203

#10 MeSH descriptor: [Nursing Care] explode all trees 2380

#11 (Management, Nursing Care OR Nursing Care Management OR Care, Nursing):ti,ab,kw 20325

#12 (Nurse Intervention):ti,ab,kw 15722

#13 #8 OR #9 OR #10 OR #11 OR #12 33694

#14 #7 AND #13 with Cochrane Library publication date Between Jan 2000 and Apr 2025, in Trials 131

Language: English, 123

## SCOPUS: 450 articles

**Search Performed on:** May 30, 2025

( TITLE-ABS-KEY ( inflammatory AND bowel AND disease* ) OR TITLE-ABS-KEY ( ulcerative AND colitis ) OR TITLE-ABS-KEY ( colitis AND gravis ) OR TITLE-ABS-KEY ( idiopathic AND proctocolitis ) OR TITLE-ABS-KEY ( crohn AND disease ) OR TITLE-ABS-KEY ( crohn's AND enteritis ) OR TITLE-ABS-KEY ( regional AND enteritis ) OR TITLE-ABS-KEY ( ileocolitis ) OR TITLE-ABS-KEY ( terminal AND ileitis ) OR TITLE-ABS-KEY ( regional AND ileitis ) OR TITLE-ABS-KEY ( granulomatous AND enteritis ) OR TITLE-ABS-KEY ( granulomatous AND colitis ) ) AND ( TITLE-ABS-KEY ( "Practice Pattern*, Nurse*" ) OR TITLE-ABS-KEY ( nurse-led AND clinic* ) OR TITLE-ABS-KEY ( nurse AND led AND clinics ) OR TITLE-ABS-KEY ( nurse AND intervention ) OR TITLE-ABS-KEY ( nursing AND care ) OR TITLE-ABS-KEY ( nursing AND care AND management ) ) AND ( LIMIT-TO ( DOCTYPE , "ar" ) ) AND ( LIMIT-TO ( LANGUAGE , "English" ) )
